# Fracture négligée du capitellum chez un adulte: à propos d'un cas et revue de la literature

**DOI:** 10.11604/pamj.2015.20.184.3978

**Published:** 2015-02-27

**Authors:** Amine Belmoubarik, Anis Achargui, Yassine Azagui, Driss Bennouna

**Affiliations:** 1 Centre Hospitalier universitaire Ibn Rochd, Casablanca, Maroc

**Keywords:** Capitellum, négligé, fracture, Capitellum, neglected, fracture

## Abstract

Les auteurs rapportent un cas de fracture du capitellum négligée chez une femme de 36 ans ayant des limitations fonctionnelles. Après revue de la littérature, ils discutent le problème de la prise en charge thérapeutique et le pronostic de la lésion.

## Introduction

La fracture du capitellum est rare. Quand elle est ancienne, elle pose un problème de prise en charge thérapeutique et de pronostic.

## Patient et observation

**Figure 1:**
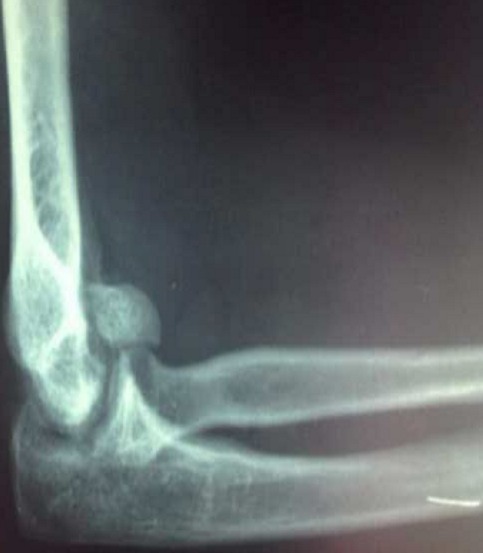
Radiographie du coude droit de profil montrant le cal vicieux du capitellum

**Figure 2:**
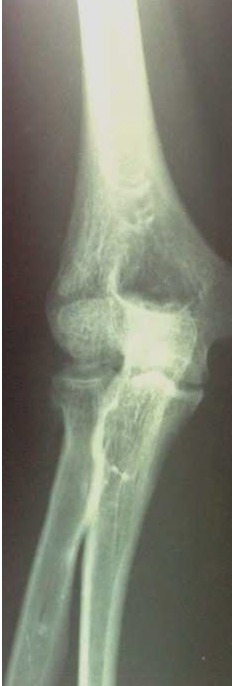
Radiographie de face du coude droit

**Figure 3:**
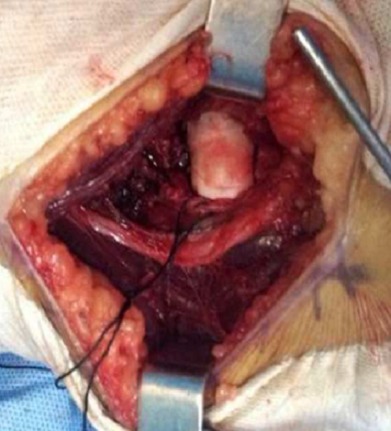
Image per opératoire montrant le fragment osseux du capitellum en position extra anatomique

**Figure 4:**
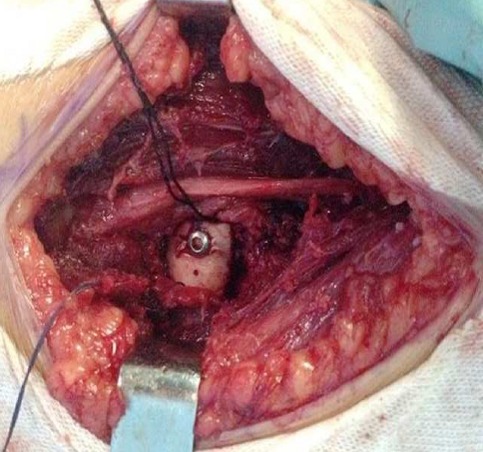
Fixation osseuse par vis après réduction

**Figure 5:**
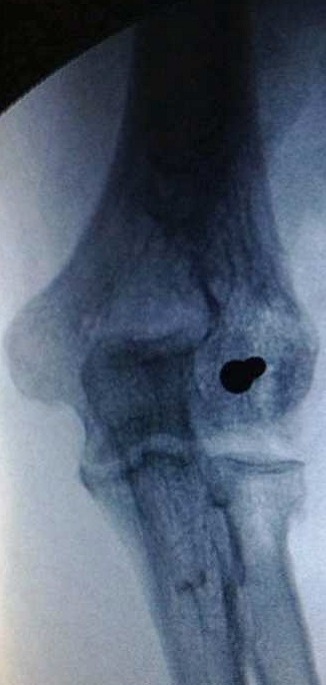
Contrôle radiographique de face du montage

**Figure 6:**
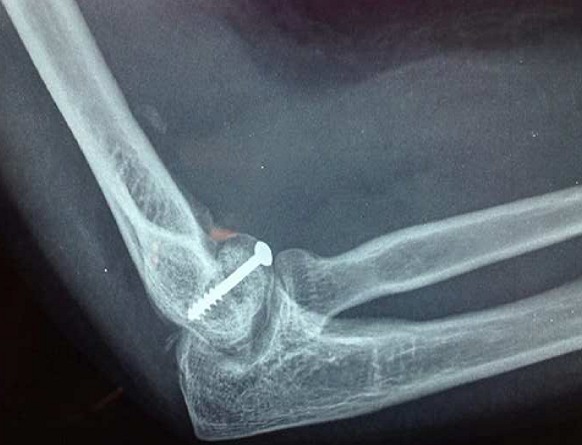
Contrôle radiographique de profil du montage

Il s'agissait de la patiente A.S. âgée de 36 ans, femme au foyer, qui rapportait un antécédent de traumatisme fermé du coude droit suite à une chute avec réception sur la paume de la main remontant à deux mois, et qui consultait pour une douleur avec limitation fonctionnelle du coude. Le coude était sensible mais pas très oedématié, les repères anatomiques du coude étaient conservés, la flexion était limitée à 95°, l'extension et la prono-supination était libres et il n'y avait pas de troubles vasculo-nerveux. La radiographie du coude gauche (face et profil) (Figure 1, Figure 2) montrait une cal vicieuse du capitellum ascensionné et en position antérohumérale sans autres lésions associées. La réduction chirurgicale du cal vicieux avait été réalisée par voie antérieure bicipitale externe, avec ostéotomie du cal, repositionnement anatomique du capitellum après préparation du site de fixation et excision de la fibrose. L'ostéosynthèse était réalisée par vissage antéro-postérieur en compression par une seule vis capitulo-humérale (Figure 3, Figure 4). La réduction était stable avec gain per opératoire sur la flexion du coude par disparition du butoir antérieur généré par le capitellum en cal vicieux (Figure 5, Figure 6). A titre antalgique, une immobilisation du coude par une attelle brachio antébrachiale pendant une semaine a été indiquée, la douleur avait disparu et la mobilité du coude était satisfaisante avec une flexion à 130°, une extension à 0°, une pronation à 90°, et une supination à 90°. Devant le caractère ancien de la lésion et surtout l’état fonctionnel postopératoire normal du coude, et la stabilité du montage nous avons préféré démarrer un protocole de rééducation fonctionnelle du coude à partir de la deuxième semaine.

## Discussion

La fracture du capitellum n'est pas fréquente, la forme négligée ou ancienne est une entité rare chez l'adulte. Les patients victimes de ce type fracture se présentent habituellement avec une douleur et un oedème du coude après un traumatisme d’énergie moyenne. Les fractures du condyle passent parfois inaperçues en première analyse, elles ne sont pas évidentes sur les radiographies de face parce que le trait de fracture n'est pas reconnu dans le contexte de l′humérus distal, c'est ainsi que Pal Singh [1] rapporte trois cas de formes négligées sur une série de quatorze fractures du capitellum. Ils sont les mieux vus sur une incidence de profil. Les radiographies doivent être soigneusement évaluées pour la recherche d'une extension médiale vers la trochlée, un refend métaphysaire et une fracture de la tête radiale ou du col qui peuvent être associés. Parfois, les radiographies sont insuffisantes et de plus en plus d'auteurs [2] recommandent le recours systématique au scanner, qui permet une étude globale de l'anatomie du coude, la recherche de lésions associées, d'une impaction articulaire mais surtout permet une planification préopératoire en ce qui concerne le choix des implants et de la voie d'abord. L’évolution naturelle d'une fracture du capitellum se fait vers la raideur du coude. En effet, un fragment du capitellum déplacé et non traité subit des changements résultant de l′absorption osseuse et la prolifération osseuse anarchique avec effacement de la fosse radiale, puis une dégénérescence arthrosique avec limitation des mouvements pour aboutir au final à la raideur du coude [3].

Plusieurs procédés de traitement de fractures du condyle ont été décrits. Il s′agit notamment de la réduction à foyer fermée, l'excision, et la réduction ouverte avec ou sans fixation interne. La Réduction fermée ne peut être proposée dans les formes négligées au vu de la fibrose qui se forme et c'est d'autant plus vrai en ce qui concerne les formes à grand déplacement. L'excision du fragment du capitellum a été décrite dans plusieurs séries [4–6] c'est un procédé simple, mais après la résection du fragment, la surface de l′os nu restant prédispose le coude à des adhérences capsulaires et à des amplitudes restreintes de mobilité, à de l'instabilité, au valgus du coude et au risque de névrite ulnaire [7]. La réduction à foyer ouvert et fixation interne (ORIF des anglo-saxons) est une méthode appropriée pour maintenir la congruence commune tout en permettant une mobilisation précoce. Les broches de Kirschner et les vis AO de compression sont les matériels les plus utilisés dans la littérature, même si la tendance actuelle penche vers l'utilisation de vis canulées et sans tête [8]. Les broches de Kirschner n′offrent pas de compression au niveau du foyer de fracture et exigent l′ablation ultérieure. La fixation par vis de l'AO répond au principal cahier de charge pour ce genre de fracture, à savoir la compression même si on peut leur reprocher une certaine irritation du cartilage de la tête radiale si la tête de vis dépasse de la surface articulaire. La voie d'abord la plus utilisée dans la littérature est une voie latérale qui permet la réduction, la fixation et l'ostéosynthèse du fragment osseux [9]. Pour notre patiente le choix de la voie d'abord antérieure, avec tous les risques décrits pour cette voie, a été motivée principalement par la variété inhabituelle du déplacement du fragment osseux en anté-huméral, rendant toute autre voie d'abord laborieuse et incertaine. La nécrose avasculaire est rare après réduction ouverte et une fixation interne de ces fractures. L′incidence déclarée de nécrose avasculaire est 0-30% [10] pour les fractures fraîches. Notre patiente n′a montré aucuns signes d′ostéonécrose malgré le fait qu′il y avait fixation des tissus mous et sans aucun support osseux important pour revasculariser le fragment. La littérature suggère que si l′ostéonécrose ne se produit pas dans un an, il est peu probable qu'elle survienne par la suite [11]. McKee et al. a rapporté un patient souffrant d′arthrose post-traumatique dans sa série de six patients [12]. Dans une grande série, Lansinger et al. n'a trouvé aucun cas d′arthrose du coude [13]. Nous n′avons pas trouvé d′arthrose chez notre patiente au dernier recul et nous croyons que si la réduction est anatomique avec un minimum de dommages au cartilage articulaire, l′arthrose post-traumatique ne devrait pas se produire.

## Conclusion

Traditionnellement, dans les formes anciennes, l'excision du fragment était recommandée en raison du risque d'ostéonécrose. Pour notre part, nous recommandons une réduction ouverte et une fixation interne avec vis pour le traitement des fractures négligées, parce que cette procédure conduit à un minimum de dommages articulaires et permet une mobilisation précoce seul garant de la conservation de la mobilité et de la stabilité du coude et ainsi de sa fonction.
